# First-in-Man Study of a Novel, Balloon-Adjustable Mitral Annuloplasty Ring

**DOI:** 10.3390/jcm13113214

**Published:** 2024-05-30

**Authors:** Paul Werner, Tandis Aref, Keziban Uyanik-Uenal, Alfred Kocher, Piergiorgio Tozzi, Guenther Laufer, Martin Andreas

**Affiliations:** 1 Department of Cardiac Surgery, Medical University of Vienna, Waehringer Guertel 18-20, 1090 Vienna, Austria; tandis.aref@meduniwien.ac.at (T.A.); keziban.uyanik-uenal@meduniwien.ac.at (K.U.-U.); alfred.kocher@meduniwien.ac.at (A.K.); guenther.laufer@meduniwien.ac.at (G.L.); martin.andreas@meduniwien.ac.at (M.A.); 2 Department of Cardiovascular Surgery, Center Hospitalier Universitaire Vaudois, 1005 Lausanne, Switzerland; piergiorgio.tozzi@chuv.ch

**Keywords:** mitral repair, adjustable annuloplasty, functional mitral regurgitation

## Abstract

**Objectives**: Mitral valve repair is the current standard approach for mitral valve regurgitation. However, patients suffering from functional mitral regurgitation have a significant risk of recurrent regurgitation. Adjustable mitral rings may provide a solution for this adverse event. **Methods**: A single-center, first-in-man clinical study was performed on patients suffering from mitral valve regurgitation. Patients were implanted with the study ring and followed for six months. A balloon catheter can be inserted into the study ring frame at any time after implantation and inflated independently in the areas P1, P2, or P3, which reduces the anterior-posterior diameter. **Results**: Five patients (75.4 ± 6.1 years; EuroSCORE II 2.1 ± 0.9%; three female) were successfully implanted. Mechanisms of mitral regurgitation were prolapse of the P2-segment in three patients and annular dilation in two patients. Surgical implantation according to the protocol was feasible and is described herein. Median cardiopulmonary bypass time and cross clamp time were 105 (118; 195) and 94 (90; 151) min, respectively. The median intensive care unit stay was 2 (2; 3) days. No perioperative, 30-day, or 6-month mortality was observed, and the repair was stable without residual or recurrent regurgitation ≥ grade 2. All patients reached the primary endpoint without device-related morbidity. **Conclusions**: Successful implantation was completed in five patients without device-related adverse events. Ring implantation was safe and feasible for all patients. The opportunity of post-implant adjustment to improve leaflet coaptation is a promising new therapeutic strategy that is assessed in a phase II study.

## 1. Introduction

Surgical mitral valve repair represents the current standard of care in patients with severe symptomatic degenerative mitral valve regurgitation (MR), standing as a Class I recommendation in current guidelines [1].

Outcomes of surgical mitral valve repair for degenerative etiology are excellent, with reports of near-normal survival in some series when surgical repair is performed timely [2]. Nevertheless, residual and recurrent MR remain a significant problem; while residual MR at discharge after the index procedure is considered a risk factor for a failing repair with reintervention [3], recurrent MR is associated with adverse ventricular remodeling and also increased mortality rates [4]. Residual MR should be avoided by choosing the correct surgical strategy, which almost always includes ring (or band) annuloplasty. Sizing of the implant plays a crucial role, as an inappropriate ring size might lead to residual MR, insufficient leaflet coaptation (which is a predictor for recurrent MR) if the ring is too large, and systolic anterior motion of the anterior mitral valve leaflet causing left ventricular outflow tract obstruction if the ring is too small. However, ring sizing is mostly performed on the arrested and non-filled heart, which can be challenging in certain anatomies and/or pathologies.

While results are univocally preferring repair over replacement in patients with degenerative mitral regurgitation, recent data questioned this approach for patients with functional mitral regurgitation [5]. Functional mitral regurgitation, except for annular dilatation due to isolated, long-standing atrial fibrillation, is caused by a ventricular disease, mainly due to ischemic or dilatative cardiomyopathy. Therefore, even after successful repair, ventricular remodeling may lead to recurrent regurgitation in patients with ischemic mitral regurgitation. This adverse event occurs in up to 30% of patients during intermediate-term follow-up after surgical mitral valve repair and is associated with increased mortality [6,7]. Although risk factors for early failure have been described, a reliable prediction of recurrent mitral regurgitation after mitral repair in patients suffering from functional mitral regurgitation is currently not possible [8]. Acker et al. described comparable outcomes for repair and replacement in this patient population. Importantly, the rate of recurrent mitral regurgitation was reduced in the replacement group [9,10]. Contrarily, valve replacement has an increased surgical risk [11]. In addition, care has to be applied to reattach the sub-valvular apparatus in patients undergoing replacement to avoid further reduction of the left ventricular function [12]. If feasible, a repair without the risk of recurrent mitral regurgitation would be the most appropriate approach in this patient population. This is underlined by the fact that the leaflets themselves are usually normal and do not require leaflet repair procedures in addition to annuloplasty.

Both clinical problems, residual and recurrent MR following mitral valve repair of degenerative or functional etiology, might be addressed by a relatively novel concept: intraoperative or post-operative correction via an adjustable annuloplasty ring. By inducing a change in ring geometry, the annular diameter is decreased, which leads to increased coaptation and a subsequent reduction of MR.

Specific rings following this novel concept were already developed and assessed in clinical trials (13 + 14). One device offered the opportunity to adjust the ring during long-term follow-up without re-opening the chest [13]. This ring had the opportunity to reduce the anterior-posterior diameter in the A2-P2 region and was based on an electrical activation mechanism of a pre-shaped nitinol ring. Another device was based on a mechanical adjustment of the ring shape under beating heart conditions intraoperatively following the implantation [14].

The novel ring applied in the current clinical study offers the opportunity to reduce the anterior-posterior distance in areas P1, P2, or P3 independently with a balloon-adjustable ring frame. We evaluated the safety and feasibility of ring implantation in this first-in-man clinical trial. 

## 2. Materials and Methods

A single-center, first-in-man clinical trial was performed at a tertiary care hospital. Our center previously participated in a clinical trial evaluating the specific treatment of patients with functional mitral regurgitation by an adjustable mitral ring [13]. Therefore, some technical and clinical aspects were anticipated and will be described in the results section. The implanting team was trained for the technical and procedural specifications in an animal lab prior to the first implantation [15]. This study was approved by the local ethical board (EC number 1471/2017) and by the competent authority (10197083).

### 2.1. Study Device

**Figure 1:**
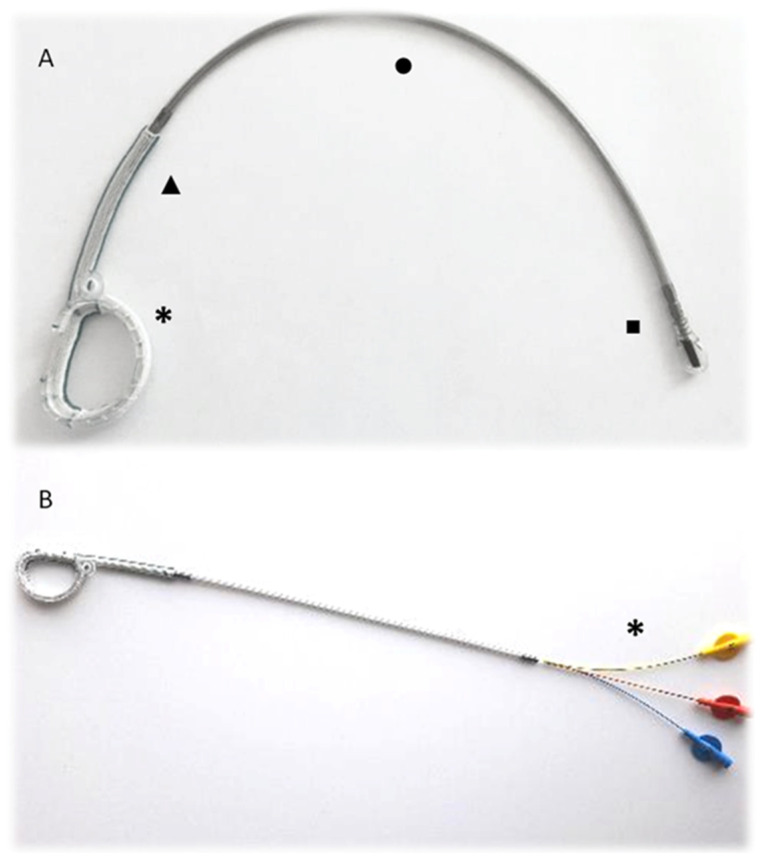
(**A**): The study device consists of a deformable annuloplasty ring (*), a proximal, textile-covered part of the connection line (▲), a distal detachable part of the connection line (●), and the long exchangeable plug mandrel inserted within the connection line for sealing (■) (**B**): A dedicated three-balloon catheter (*) for post-implantation adjustment is inserted within the connection line.

**Figure 2:**
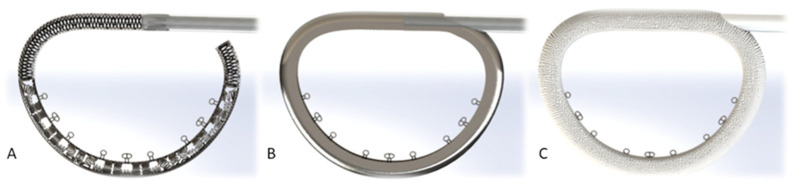
Structural design of the annuloplasty ring from inside to outside (**A**): deformable cage with inner-sided eyelets for suture placement (**B**): externally rigid ring surrounding the deformable cage (**C**): textile fabric sleeve covering all parts of the ring except suture eyelets.

**Figure 3:**
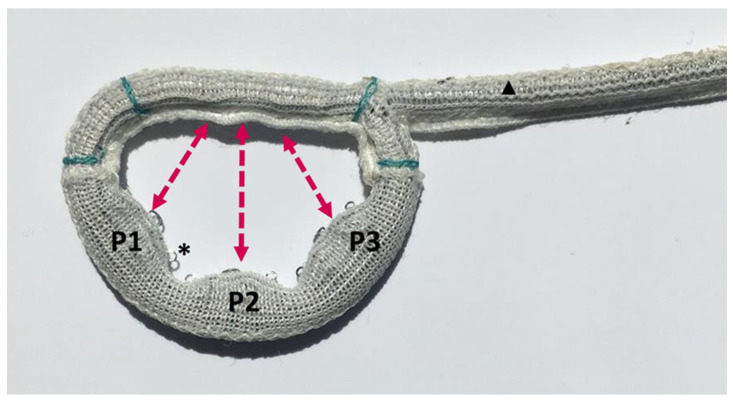
KD1 Device with posterior ring segments in fully expanded position; * indicating eyelets for suture placement on the posterior aspect of the ring; ▲ indicating the proximal part of the connection line, allowing the insertion of a balloon-catheter for ring adjustment.

The KD1 ring (Affluent Medical, Aix-en-Provence, Provence-Alpes-Côte d’Azur, France) is an adjustable, D-shaped, flat mitral annuloplasty ring (Figure 1A). Its structure is hollow and consists of an external, rigid, and closed ring (Figure 2A), which surrounds a deformable, D-shaped cage (Figure 2B) covered by a textile fabric sleeve (Figure 2C). The KD1 can be adjusted at any time after surgical implantation by expanding the deformable cage inwardly into the valve lumen by means of a dedicated three-balloon catheter actuator (Figure 1B). This catheter is introduced into the hollow structure of the ring through a connection line (a plastic tubing), which connects the ring via passage through the caudal end of the left atrial atriotomy (Supplementary Materials Figure S1) to a subcutaneous position. It is preferably routed in the epigastric area using a long clamp or similar instrument. The connection cable might also be positioned under the rectus abdominis muscle in very thin patients. If there are any contraindications against positioning the device in the epigastric area, a routing in the subclavian subcutaneous position is also possible. The geometry of the ring can be adjusted independently in the three portions corresponding to the three scallops of the posterior mitral valve leaflet known as P1, P2, and P3 under beating heart conditions using the three-balloon catheter (Figure 3). Multiple adjustments, either during the same procedure or in several procedures at different times, can be performed. Adjustment is performed under fluoroscopy, which aids the insertion depth of the balloon catheter until the desired segment (additionally, color-coded markers on the outside are also indicating the depth of the desired segment). Device adjustment is then performed under fluoroscopic and echocardiographic guidance. Fluoroscopy is helpful in showing the extent of the expansion of the desired scallop, whereas echocardiographic live imaging is monitoring the dynamic of the regurgitant jet. Additionally, parameters such as coaptation length or tethering area can be measured before and after the adjustment. The effect of the balloon inflation on the geometrical change of the ring has already been reported by our group during the course of a preclinical animal trial of the study device [15].

### 2.2. Patients

Patients referred to our center for repair of mitral valve regurgitation were invited to participate in this clinical trial. Inclusion criteria were patients with symptomatic severe mitral regurgitation (Carpentier’s classification Type I or II P2) or asymptomatic patients with preserved left ventricular function and new onset of atrial fibrillation or pulmonary hypertension; with an EuroScore II < 4; with left ventricular ejection fraction ≥ 55%; with a normal coronary angiogram (no significant lesions); in satisfactory condition, with a life expectancy above one year; able to understand and willing to sign the informed consent; and willing to undergo planned follow-up visits. Patients were excluded if they were under 18 years; pregnant or nursing; requiring a MRI (magnetic resonance imaging) examination; involved in any other clinical investigation for drugs or devices; with previous cardiac surgery or diaphragmatic lesion or previous hepatic surgery; needing an acute intervention; with active endocarditis (or having had active endocarditis in the last three months); with active myocarditis; with heavily calcified mitral annulus or a mitral valve anatomy with a high risk of valve replacement instead of valve repair; needing any cardiac surgery other than mitral repair, tricuspid valve annuloplasty, pacemaker implantation (epicardial), exclusion of left atrial appendage (with device or surgically) and a maze- or pulmonary-vein isolation procedure; with severe pulmonary hypertension (systolic pulmonary artery pressure at rest > 65 mmHg); with creatinine level > 2.0 mg/100 mL; with echocardiographic measurements predicting SAM (systolic anterior motion of the mitral valve); unable to take anticoagulation medications; with a known untreatable allergy to contrast media or nickel; with a major or progressive non-cardiac disease that, in the investigator’s experience, results in a life expectancy shorter than 1 year, or for whom the implant of the device poses an unacceptable increase of risk; having had an acute preoperative neurological deficit, myocardial infarction, or cardiac event that has not returned to baseline or stabilized ≥ 30 days prior to the planned surgical procedure; unable to understand and sign the informed consent form; unable to read and write or with an anticipated ring size very small (26 mm) or very large (36 mm).

### 2.3. Clinical Trial

**Figure 4:**
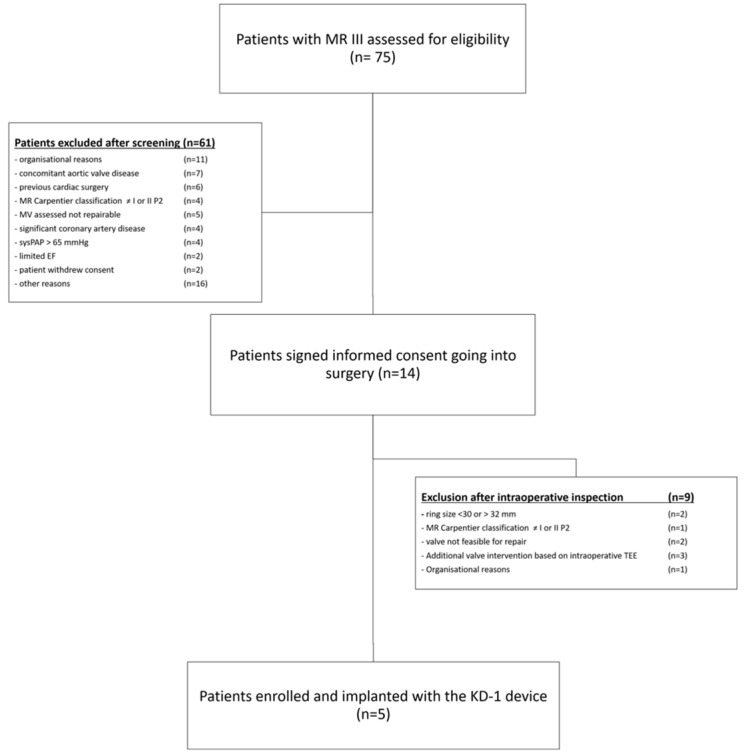
Consort flow diagram of the screening and enrolment process of the OPTIMIZE I trial. MR = Mitral regurgitation, MV = Mitral valve, sysPAP = Systolic pulmonary artery pressure, EF = Ejection fraction.

Patients were included in the trial after signing a written informed consent. A scientific advisory board supported the local study team to clarify questions regarding the suitability of patient inclusion. The ring was available in two sizes (30 mm and 32 mm). Therefore, patients who consented to this study but required another ring size were implanted with a different ring and not followed by this protocol (Figure 4). A clinical examination including NYHA (New York Heart Association) class, laboratory tests, electrocardiography, chest X-ray, and transthoracic echocardiography (TTE) were performed at inclusion, during the postoperative hospitalization or at discharge, at 30 days, at three months, and at six months after surgery.

### 2.4. Statistical Analysis

Descriptive statistical methods were applied to depict the study population. The primary objective was defined as safety in terms of mortality and morbidity at 30 days after implantation (primary endpoint) and at three and six months (secondary endpoint). Furthermore, reduction of mitral regurgitation, coaptation length, and absence of recurrent mitral regurgitation were assessed. Continuous variables were presented as mean and standard deviations or median (25th–75th interval). Total numbers and proportions were reported for categorical outcomes. An analysis of variance was performed to assess changes in specific echocardiographic parameters. A *p*-value of less than 0.05 was considered statistically significant. IBM SPSS Statistics 24 (IBM Corp. released 2016). IBM SPSS Statistics for Mac, Version 24.0 (IBM Corp., Armonk, NY, USA) was used for statistical analysis.

## 3. Results

Seventy-five patients were screened, and 14 patients signed the informed consent form, of which nine were excluded intraoperatively due to the necessity of additional valve interventions based on the intraoperative TEE (trans-esophageal echocardiography) (n = 3), a required ring size smaller than 30 mm or greater than 32 mm (n = 2), a mitral valve deemed not feasible for reconstruction (n = 2), a Carpentier’s classification other than I or II P2 (n = 1), and organizational reasons (n = 1) (Figure 4—Consort Flow Diagram). Five patients (75.4 ± 6.1 years; EuroSCORE II 2.1 ± 0.9%; three female) were deemed eligible during surgery and successfully implanted with the study device. Patients were rather low-risk without concomitant bypass surgery or aortic valve procedures due to the inclusion/exclusion criteria (Table 1). Mechanisms of mitral regurgitation were prolapse of the P2-segment in three patients and annular dilation in two patients. 

**Figure 5:**
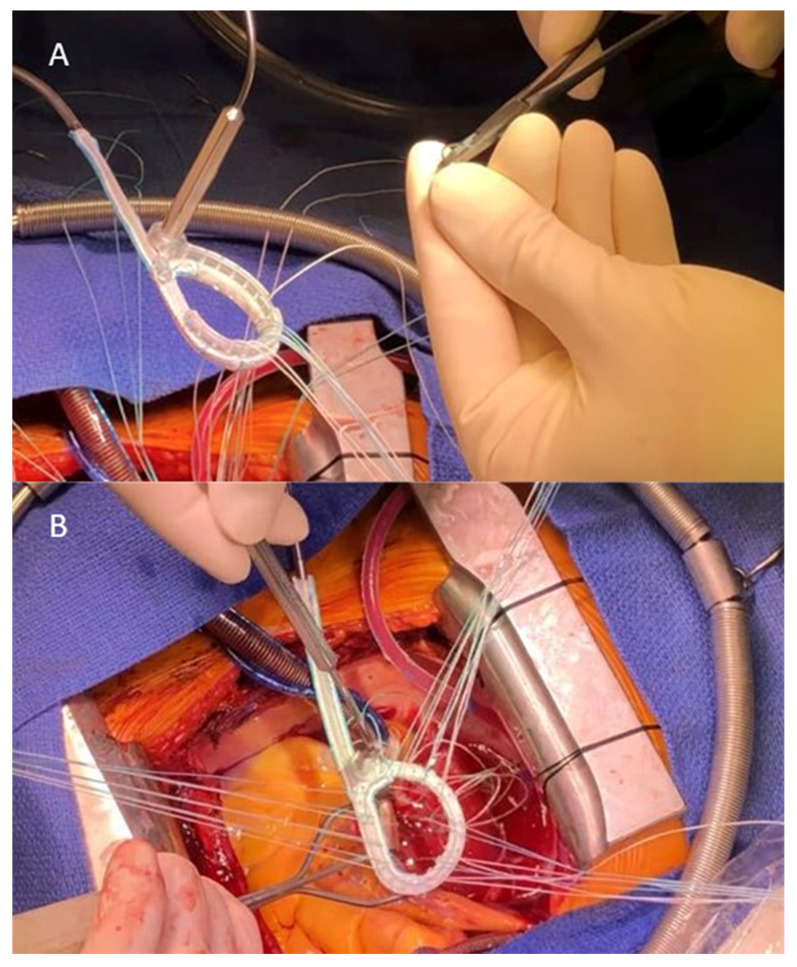
(**A**) Suture placement through specialized eyelets on the inner side of the posterior ring segment; (**B**) Lowering of the ring after suture placement through the eyelets.

Surgical access was a median sternotomy in all patients. Three patients underwent isolated mitral valve repair, and two patients underwent mitral valve repair with concomitant procedures (Table 2). Four patients received a 30 mm ring, and one patient received a 32 mm ring. Additional leaflet repair procedures were performed in three patients (Table 2). Surgical implantation of the study ring was feasible without revisions or adverse events. A crucial step during implantation was the suture placement, which had to be performed according to the position of the eyelets on the posterior ring segment (Figure 5A, Video S1). Sutures were then passed through the inside of the ring (Figure 5B). Furthermore, due to the direct contact of the suture with the metal eyelet, the sutures were crossed twice during knotting to improve knot stability, as the tying force was carefully reduced to avoid bending the eyelets prior to adjustment. The routing of the connection cable was performed through the caudal end of the left-sided atriotomy in all cases and proved to be uneventful in every patient. It was fixed with a 4-0 polypropylene suture to the inner side of the left atrium and again at the caudal end of the incision with additional pledgeted sutures. These were placed solely for hemostatic reasons.

Median cardiopulmonary bypass and cross clamp times were 105 (118; 195) and 94 (90; 151) minutes, respectively (Table 2). No perioperative, 30-day, or 6-month mortality was observed. The median ICU (intensive care unit) and hospital stay were 2 (2, 3) and 11 (7, 19) days, respectively. All patients reached the primary endpoint with no mortality or device-related morbidity and were discharged home. Mitral regurgitation improved from severe regurgitation (n = 5) at the baseline visit to non/trace (n = 4) or mild regurgitation (n = 1) at the 6-month follow-up visit. No moderate or severe recurrent mitral regurgitation was observed during the 6-month follow-up period (Table 3). 

Echocardiographic parameters were compared perioperatively, after implantation, and at 6-month follow-up. Coaptation length increased numerically from three (±1) mm before implantation to five (±1) mm after implantation and at 6-month follow-up (*p* = 0.053). Ejection fraction, LVEDD (left ventricular end diastolic diameter), and LVESD (left ventricular end systolic diameter) did not change over time (Table 3).

In one patient, a pacemaker lead revision and a postoperative cardioversion due to pre-existing atrial fibrillation were required, and he suffered from a urinary-tract infection. One protocol deviation occurred in a different patient with severe tricuspid regurgitation who was planned for tricuspid repair but needed to undergo tricuspid replacement after a failed repair during the same procedure. The underlying mechanism was a pacemaker lead, which perforated the papillary muscle and prevented successful repair due to significant sub-valvular changes. (The protocol deviation was reported to the responsible ethical committee and accepted). The same patient suffered from sternal bleeding requiring rewiring, intermittent dialysis, pacemaker lead extraction, and a deep sternal wound infection requiring reconstructive surgery. No adverse events were observed in the other three patients.

## 4. Discussion

Five patients received a novel, adjustable mitral ring in this first-in-man clinical trial. The technical details specifically required to implant this device were previously published in an animal model and confirmed in this clinical trial [15]. No device-related adverse events were observed, and mitral valve repair was stable throughout the study period. The primary goal of this trial was to assess the safety and feasibility of implantation. Therefore, the patient population was defined as a low-risk cohort without reduced ejection fraction or the presence of functional mitral regurgitation (Carpentier IIIb). Hence, we did not expect to observe any conditions requiring ring adjustment, which was not performed in this trial. 

A comparison of mitral leaflet coaptation length directly before and after device implantation and at 6-month follow-up showed a numerical increase in coaptation length. This parameter is an important measurement after mitral valve reconstruction and a predictor of recurrent MR [16]. Regular echocardiographic observation of the coaptation length might benefit patients after KD1 implantation in order to initiate timely postoperative ring adjustment to prevent the recurrence of MR.

Functional mitral regurgitation is a significant predictor of a worse outcome in patients with cardiomyopathy [17]. Despite this fact, current guidelines are very cautious when recommending surgical or interventional procedures for this patient population. This may not be related to the disease itself but more to the lack of adequate and durable treatment options for this complex pathology. 

Recent trials did not show a significant difference regarding survival for mitral valve repair or replacement in patients suffering from functional mitral regurgitation [9,18]. Acker et al. revealed a numerically higher perioperative mortality after replacement but reported an increasing rate of recurrent mitral regurgitation in the repair group. This may translate into a higher rate of reoperations, which was previously highlighted by Lorusso et al. [18]. As previously reported by our group, adjustable mitral rings may present an alternative to current treatment options [13]. Recurrent mitral regurgitation after repair could be treated with a minor, subcutaneous procedure, avoiding re-operation and allowing a treatment adaptation to ventricular remodeling. This would also avoid the higher perioperative mortality reported for mitral valve replacement. However, patient selection is key, and specific characteristics like inferobasal aneurysms, severe leaflet tethering, or a severely dilated left ventricle may indicate initial valve replacement [19]. 

A variety of disease-specific rings were developed to treat functional mitral regurgitation [14,20,21]. While passive rings have a specific shape to adapt the annulus for ventricular remodeling, adjustable rings can be modified during surgery or even long-term after surgery by a minor procedure. The main concept was the reduction of the anterior-posterior diameter, or annular circumference. Czesla et al. reported the first successful adjustment with another system three months after implantation [22]. We previously implanted an adjustable ring in a larger cohort, including functional mitral regurgitation, and observed some improvement after ring adjustments [13]. The novel ring studied herein allows a more tailored adjustment according to the specific area of regurgitation. The ring is based on a previous design, allowing adjustment with a positionable spacer, which was iterated into a balloon-adjustable concept for the current trial [23]. The adjustment can be adapted to the regions P1, P2, and/or P3 under 3D echocardiographic guidance. During adjustment, the inward movement of the annulus will increase coaptation and reduce regurgitation. The tailored adjustment may be a crucial improvement compared with previous concepts, allowing personalized postoperative correction of a regurgitant jet in a specific location. This single segment targeting might allow for correction of the residual/recurrent MR without induction of the form change in the other segments, thereby reducing the chance of an unwanted transvalvular gradient increase.

This therapeutic device might offer a novel concept to tackle residual or recurrent MR in patients after surgical reconstruction. However, one must be aware of possible complications arising from the novel study device design. Sutures on the posterior ring segments are placed through the metal eyelids and might be subject to increased mechanical stress during ballon inflation with scallop deformation. To mitigate this problem, suture tying is performed with double crossing of the sutures to increase knot stability. We believe that the mitral annulus retains a certain pliability; therefore, induction of a geometrical form change of the posterior ring segments with displacement of the eyelids of only 1–3 mm might not result in ring dehiscence.

The placement of the subcutaneous connection line offers reliable access for postoperative adjustments. Nevertheless, infection of this structure bears the risk of prosthetic endocarditis of the ring itself, as they are connected. Therefore, following a perioperative adjustment, the connection line can be exchanged with a shorter plug. This will be positioned within the pericardium and not subcutaneously. In cases of connection line infection, a combination of topical and systemic antibiotics with surgical shortening of the connection line might be beneficial (similar to pacemaker leads, silicon caping of the connection line might be performed). In the case of an infected mitral ring prosthesis, surgery should be performed with acceptable surgical risk.

### Limitations

The current trial is a small, phase I, first-in-man trial, not assessing the device adjustment procedure itself. It consists of a low surgical risk collective with patients suffering only from degenerative mitral regurgitation, not assessing the ring in patients with secondary mitral regurgitation. Based on the fact that this is a phase I trial assessing a permanent intracardiac device, the decision against the inclusion of the less healthy, comorbidity-burdening collective of FMR patients was made, as these patients are investigated in a phase II clinical trial.

## 5. Conclusions

Successful implantation was completed in five patients without device-related adverse events. Ring implantation was safe and feasible for all patients. The opportunity for post-implant adjustment to improve leaflet coaptation is a promising new therapeutic strategy that is currently being assessed in a phase II trial.

## Figures and Tables

**Table 1:** Patient characteristics at the time of the procedure (n = 5).

Characteristics	Values
Age (years)	74.4 (±6.2)
Height (cm)	170.0 (±15.8)
Weight (kg)	73.0 (±18.9)
BMI (kg/m^2^)	24.8 (±2.1)
NYHA class	2 (2; 2)
EuroScore II (%)	2.1 (±0.9)
Risk factors	
Smoking	0 (0%)
Diabetes	1 (20%)
Dislipidemia	2 (40%)
Comorbidities	
Atrial Fibrillation	3 (60%)
Severe TR	2 (40%)
sysPAP (mmHG)	49.0 (±8.4)

Caption: Values are mean (±SD), median (Q_1_, Q_3_), or N (%); BMI = Body Mass Index; NYHA = New York Heart Association; sysPAP = systolic pulmonary artery pressure; TR = tricuspid regurgitation.

**Table 2:** Procedural characteristics of mitral valve reconstruction.

Procedural Data	Values
Ring size 30 mm	4 (80%)
Ring size 32 mm	1 (20%)
Artificial chordae	2 (40%)
Triangular resection	1 (20%)
Leaflet plication	1 (20%)
Procedural times (min)	
Surgical time	260 (276; 389)
CBP time	105 (118, 194)
ACC time	94 (90, 150)
Concomitant procedures	
Tricuspid repair/replacement	2 (40%)
Atrial fibrillation surgery	2 (40%)

Caption: Values are mean (±SD); median (Q_1_, Q_3_), or N (%); ACC = aortic cross clamp; CBP = cardiopulmonary bypass.

**Table 3:** Pre- and postoperative echocardiographic data.

Echocardiographic Measurements	Pre-Implant (TEE)	Post-Implant (TEE)	6-Month FU (TTE)	*p*-Values
EF (%)	66 (±8)	52 (±1)	59 (±5)	0.151
LVEDD (mm)	51 (±6)	53 (±7)	41 (±19)	0.440
LVESD (mm)	36 (±6)	39 (±11)	32 (±4)	0.494
Coaptation length (mm)	3 (±1)	5 (±1)	5 (±1)	0.053

Caption: Values are mean (±SD); median (Q_1_, Q_3_), or N (%); FU = follow-up; EF = ejection fraction; LVEDD = left ventricular end diastolic diameter; LVESD = left ventricular end systolic diameter; TEE = transesophageal echocardiography; TTE = transthoracic echocardiography.

## Data Availability

The raw data supporting the conclusions of this article will be made available by the authors on request.

## Supplementary Materials

The following supporting information can be downloaded at: https://www.mdpi.com/article/10.3390/jcm13113214/s1. Video S1. Device description and implantation procedure of a novel adjustable mitral annuloplasty ring; Figure S1: Intraoperative image of the study device in implanted position with the prefinal routing of the connection line (indicated by asterix *) at the caudal end of the left atrial atriotomy.
