# Supplementary figures and images for: First-in-Man Study of a Novel, Balloon-Adjustable Mitral Annuloplasty Ring
Source: J Clin Med. 2024 May 30;13(11):3214. doi: 10.3390/jcm13113214 (PMC11172768; doi:10.3390/jcm13113214)

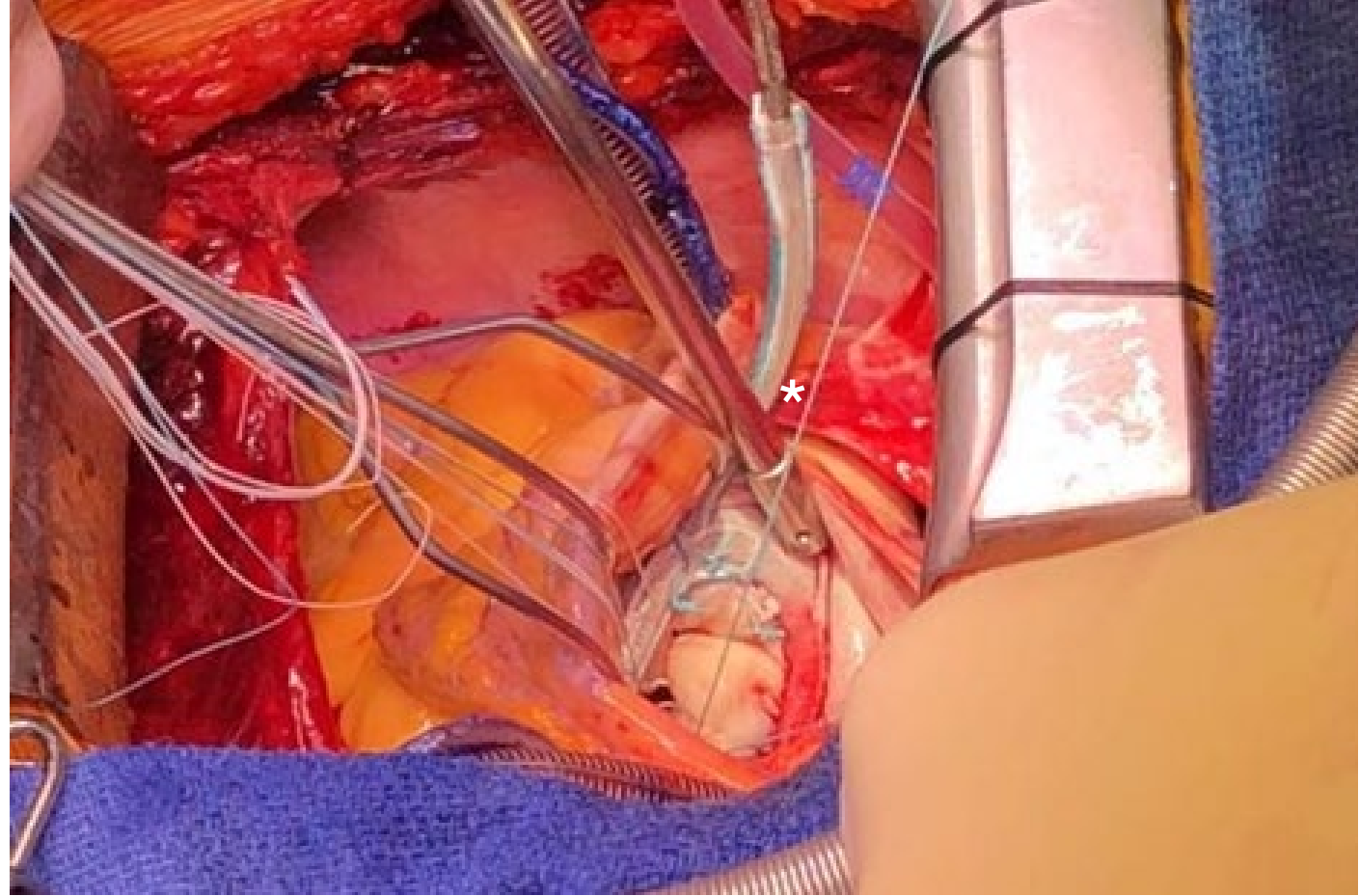

Supplement: Supplementary file 1 [file jcm-13-03214-s001.zip › jcm-2978102-Figure S1.pdf]
